# Uncertainties in the path to 2030: Increasing trends of under-five mortality in the aftermath of Millennium Development Goal in Eastern Ethiopia

**DOI:** 10.7189/jogh.12.04010

**Published:** 2022-01-22

**Authors:** Merga Dheresa, Hirbo Shore Roba, Gamachis Daraje, Mesfin Abebe, Abera Kenay Tura, Tesfaye Assebe Yadeta, Yadeta Dessie, Tariku Dingeta

**Affiliations:** 1 School of Nursing and Midwifery, College of Health and Medical Sciences, Haramaya University, Harar, Ethiopia; 2 Hararghe Health and Demographic Surveillance Systems, Harar, Ethiopia; 3 School of Public Health, College of Health and Medical Sciences, Haramaya University, Harar, Ethiopia; 4 Department of statistics, College of Computing and Informatics, Haramaya University; 5 Department of Obstetrics and Gynaecology, University Medical Centre Groningen, University of Groningen, the Netherlands

## Abstract

**Background:**

Although Ethiopia was applauded for achieving the Millennium Development Goal (MDG) target of reducing child mortality, whether the gains sustained beyond the MDG era was rarely studied. In this study, we reported the trends and determinants of under-five mortality (U5M) from 2015 to 2020 in a population based cohort under the Kersa Health and Demographic Surveillance System (HDSS), eastern Ethiopia.

**Methods:**

We followed pregnant women and their pregnancy outcomes from 2015 to 2020. Each year, data related to death and live births among the follow up population was retrieved. Automated verbal autopsy (InterVA-4) was used to assign the cause of death and Stata 14 was used for analysis. U5M rate was calculated as death among under five children divided by all live births during the study period and described per 1000 live births along with 95% Confidence Interval (CI). A multivariable Cox proportional regression model was used to identify determinant of U5M using adjusted hazard ratio (AHR). Finally, *P* value <0.05 was considered for declaring statistically significant association.

**Results:**

From January 2015 to December 2020, a total of 28 870 live births were registered under the Kersa HDSS, of whom 1335 died before their fifth birthday. The overall U5M rate was 46.3 per 1000 live births (95% confidence interval (CI) = 43.79-48.79), with significant increase from 27.9 in 2015 to 54.7 in 2020 (*P* < 0.041). Diarrheal diseases, acute respiratory tract infection including pneumonia, meningitis and encephalitis, and HIV related deaths were the leading causes of U5M. The hazard of death was higher among children born to poor household (AHR = 1.52; 95% CI = 1.27-1.81), rural residents (AHR = 6.0; 95% CI = 3.65-9.91), born to adolescent mothers (AHR = 1.41; 95% CI = 1.02-1.95), whose mother didn’t receive antenatal care (AHR = 1.43; 95% CI = 1.21-1.69), were born preterm (AHR = 14.1; 95% CI = 9.96-19.89) and had low birth-weight (AHR = 1.74; 95% CI = 1.39-2.18).

**Conclusion:**

We found high level of U5M rate with an increasing trend in the aftermath of the praised MDG4 achievement. Achieving the ambitious U5M of 25 per 1000 live births by 2030 requires addressing diarrheal disease, and respiratory tract infections, and HIV/AIDS. Reasons behind the persistent increase over the study period require further inquiry.

Over the last three decades, substantial global progress has been made in reducing under-five mortality rate (U5MR) by 59%, from 12.6 million in 1990 to 5.2 million in 2019. However, sub-Saharan Africa and Central and Southern Asia which are home to only 52% of the under-five population account for more than 80% of the 5.2 million under-five mortality (U5M) in 2019 [1,2]. For example, half of all the U5M in 2019 occurred in just five countries: Nigeria, India, Pakistan, the Democratic Republic of the Congo, and Ethiopia. In sub-Saharan Africa, one in every 13 children dies before reaching their fifth birthday [1]. Although Ethiopia is one of the countries which achieved the millennium development goal (MDG) targets of reducing U5M by two-third, the country is still home to high burden of U5M in Africa (third) and globally (tenth). Achieving the Sustainable Development Goals (SDG) 3.2.1 target of U5M rate to 25 per 1000 live births by 2030 requires understanding of the trends and determinants of mortality for designing appropriate interventions [3].

Moreover, the gains in reducing the U5MR were not equitable between and within regions. For example, the U5MR in Ethiopia in 2019 ranged from 29 in Addis Ababa to 74 per 1000 live births in Afar Region [4]. This inequity gap remains a challenge to achieve SDG targets, specifically among more marginal populations and rural settings [5]. A study by Nick et al. showed that the key factors to meet SDG targets at the national level are political commitment, financial support, and medical advances [6]. Recent analyses of the Ethiopian Demographic and Health Survey (DHS) 2016 data showed that several socio-demographic characteristics, access and quality of maternal health care services, infections, birth interval, and breastfeeding conditions were the major predictors of U5M [7-9]. These factors may not have similar effects given that there are substantial regional and sub-regional variations.

Health and demographic surveillance systems (HDSS) emerged as the best source of representative data for evidence generation, especially in rural settings and countries with no (weak) vital registration systems. The importance HDSS in responding to public health problems have been evident with the emergence of several DSS sites since the establishment of the first HDSS site – MATLAB – in 1966 [10]. Unlike facility-based studies or Demographic and Health Surveys, HDSS provides longitudinal data of a known and stable target population that enable us to understand trends and changes in the magnitude of determinants of mortality for tailored interventions. Unfortunately, studies in Ethiopia mainly used DHS data and provide a one-time estimate of U5MR than overtime changes with little information regarding the cause of death. Others are facility-based studies with limited applicability due to lack of appropriate denominators and being limited to urban centers. Hence, there is heightened need for more evidence that can show the change of U5MR over time and the major cause of deaths. Therefore, this study was intended to identify trends and causes of U5M in Kersa HDSS from 2015 to 2020.

## METHODS

**Figure 1:**
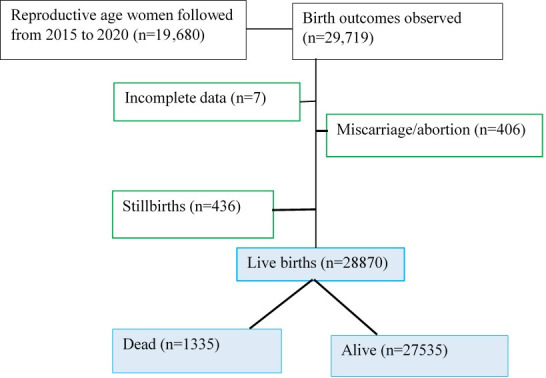
Flowchart of pregnancy observation and under-five deaths from 2015 to 2020 in Kersa HDSS, Eastern Ethiopia.

We used data from the ongoing Kersa HDSS, an HDSS affiliated with the College of Health and Medical Sciences, Haramaya University, eastern Ethiopia. Kersa HDSS is composed of two HDSS sites covering rural and urban settings: Kersa District and Harar Town. Kersa HDSS, established in 2007 in 12 kebeles (smallest administrative units in Ethiopia with 1500 average households) and added another 12 kebeles in 2015, was a predominantly rural HDSS [11]. In 2012, the HDSS field site was extended to Harar Town, a predominantly urban HDSS, covering six kebeles. Similarly, the Harar HDSS doubled its coverage to include 12 kebeles in total in 2015. Hence, Kersa HDSS covers 36 kebeles: 24 rural and 12 urban kebeles with a total population of 197 268 in 41 056 households [12]. All individuals who are living in HDSS were visited twice a year and individual updates are recorded and entered into the database system. The data was retrieved from all data generated from 2015 to 2020 as part of the routine HDSS data, including verbal autopsy data for all deaths during the same period. From a total of 29 719 birth outcomes during the study period, 406 were miscarriage/abortion and 436 were stillbirth. Thus, we followed 28 870 live births in the HDSS during the study period (**Figure 1**).

### Study design and population

Kersa HDSS is an open dynamic cohort designed to capture longitudinal records of health and demographic data to monitor vital events – births, deaths, and migration in a stable population at regular intervals – and updates relevant changes biannually [12]. Data were collected using ODK by well-trained regular HDSS staffs through face-to-face interview. Field supervisors checked data quality using GPS coordinates, consistency, and validity of the response before it was sent to the Open HDSS database. If supervisors found a data quality problem, the data collectors were redeployed for correction under direct supervision. All collected data was temporarily stored on ODK aggregate. Then, the data manager reviewed the quality of data in ODK and migrated the data from temporary storage to the final Open HDSS database.

In this study, we followed pregnant women and respective pregnancy outcomes from 2015 to 2020. Data related to deaths and live births among the follow up population was retrieved for each year together with socio-demographic characteristics, household information, prenatal cares, obstetric characteristics, and child health conditions from birth to outcome of event (death) or censored (out migrated and alive). In addition, verbal autopsy was performed for each death through interview of the mother or the closest family member within 45 days of death of the child as described elsewhere [13]. Then, the verbal autopsy information was transferred to the automated verbal autopsy (InterVA-4) algorithm for assigning cause of deaths [14].

### Measurement and variables

Women who gave birth were interviewed about outcome of their pregnancy (live births, stillbirth, or miscarriage) or any deaths of household members every six months. If death was reported, detailed information regarding the age of the deceased, date of death, sex, perceived cause of death, and place of death was collected through redeployment of data collectors using the standard World Health Organization verbal autopsy questionnaire [15,16]. The outcome variable was then categorized as ‘death’ or ‘alive’. Death was assigned “1”, and alive (censored) was a “0”. Socio-demographic factors (place of residence, education status of mother, wealth index), maternal factors (age at first birth), and obstetrics factors (parity, place of delivery, ANC visits), child related factors (birth size, estimated gestation, and sex) were considered as explanatory variables for the U5M.

### Ethical considerations

Kersa HDSS has obtained ethical clearance from the Science and Technology Minster of the Federal Democratic Republic of Ethiopia national ethical review committee (Ref No. EPHA/OG/1861/15) and the Institutional Health Research Ethical Review Committee (IHRERC) of College of Health and Medical Sciences, Haramaya University (Ref No and IHRERC/271/2014). Anonymity of data was maintained throughout the research process. All study participants gave informed written consent before inclusion into the HDSS.

### Data management and statistical analysis

Data captured using ODK was exported to Stata 14 (Stata Corp 2015, College Station, TX) for analysis. U5MR was calculated as the death of a child during the first five years of life from all the live births during the same period and expressed per 1000 live births. Neonatal mortality rate was also calculated by neonatal death from all livebirths during the study period per 1000 live births. Kaplan-Meier survival curve was used to show patterns of death in 5 years, and a log-rank test was used to compare the survival curves among independent variables. Multivariable Cox proportional regression model, adjusted for socio-demographic characteristics (maternal education, and residence), reproductive and maternal health variables (parity, receive antenatal care, place of delivery, age at first birth childbirth), neonate related variables (birth weight, gestational age, and sex of newborn) was fitted to identify predictor variables. We consider the tests of equality across strata to include the predictor in the final model. For the categorical variables we used the log-rank test of equality across strata, and for the continuous variables we used a univariate Cox proportional hazard regression model. We consider the predictors if the test has a *P* value of <0.25. Moreover, we checked proportionality assumption by including time-dependent covariates in the model. The results were reported using adjusted hazard ratio (AHR) with 95% confidence interval (CI). We checked multi-collinearity using variance inflation factors (VIF). We exclude gravidity from the model for it has collinearity with parity (VIF = 10.41). In the multivariable analysis, variables having *P* value <0.05 were considered as significant predictors of mortality.

## RESULTS

### Socio-demographic characteristics

From 2015 to 2020, we followed 19 685 pregnant women and 29 719 birth outcomes: 28 870 livebirths, 413 miscarriage/abortion, and 436 stillbirths. Majority of the women were rural residents (23 714; 82.1%), older than 24 years (20 583; 72%), and were housewives (22 688; 78.8%) (**Table 1**).

**Table 1:** Characteristics of study participants in Kersa HDSS from 2015 to 2020, eastern Ethiopia

Variables	Frequency	%
Residence (n = 28 870):		
Harar Town	5156	17.86
Kersa	23 714	82.14
Mother age (n = 28 870):		
12-19	2031	7.10
20-24	5993	20.95
25-35	14 959	52.29
35-49	5624	19.66
Ethnicity (n = 28 776):		
Oromo	25 409	88.30
Amhara	2089	7.26
Other	1278	4.44
Educational status (n = 28 870):		
Literate	11 241	38.94
Read and or write	518	1.79
Neither read nor write	17 111	59.27
Occupation (n = 28 790):		
Housewife	22 688	78.81
Daily laborer	622	2.16
Merchant	943	3.28
Unemployed	3561	12.37
Paid employer	976	3.39
Wealth index (n = 27 775):		
Poor	9438	33.98
Middle	9490	34.17
Rich	8847	31.85
Age at first child birth (n = 28 801):		
Less than 20 years	17 446	60.57
20 years or older	11 355	39.43
ANC (n = 28 870):		
Yes	13 343	46.22
No	15 527	53.78
Place of child birth (n = 28 870):		
Home	16 644	57.65
Health facility	12 226	42.35
Birth attendant (n = 28 869):		
TBAs	12 850	44.51
Health professional	11 968	41.46
Relative/ neighbors	4051	14.03
Term of pregnancy (n = 28 844):		
Term	27 021	93.68
Preterm	286	0.99
Post-term	1537	5.33
Birth weight (n = 28 651):		
Low	2212	7.72
Normal	23 316	81.38
Big	3123	10.90
Gender of child (n = 28 870):		
Female	13 599	47.10
Male	15 271	52.90

TBA – traditional birth attendants HDSS – Health and Demographic Surveillance System

### Trend of under-five mortality

**Figure 2:**
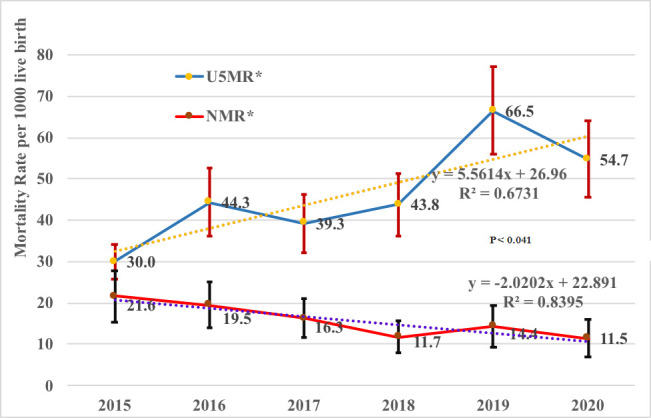
Trends of neonatal and under 5 mortality rates per 1000 live births in Kersa HDSS, eastern Ethiopia (n = 1335). U5MR – under-five mortality rate, NMR – neonatal mortality rate.

A total of 1335 children died before their fifth birthday. More than a third (447; 33.5%) of the deaths were neonates making the NMR 46.3 per 1000 live births. Majority of the deaths occurred at home (80%; 95% CI: 78%-82%). The overall U5MR was 46.3 per 1000 live births (95% CI; 43.79-48.79). The annual mortality rate significantly increased from 27.9 per 1000 live births in 2015 to 54.7 per 1000 live birth in 2020 (*P* < 0.041). The highest (66.4 per 1000 live births) U5MR was in 2019 (**Table 2**). In contrast to the trend of under-five mortality rate, the neonatal death rate showed decreasing trend (*P* < 0.04) (**Figure 2**).

**Table 2:** Under five mortality rates from 2015-2020 in Kersa HDSS Eastern Ethiopia

Year	Live births	Under-five death	U5MR/1000 LB (95% CI)
2015	3906	109	27.91 (29.13-33.66)
2016	4785	212	44.31 (38.54-50.68)
2017	5422	213	39.28 (34.18-44.92)
2018	5502	241	43.80 (38.44-49.69)
2019	4560	303	66.44 (59.17-74.36)
2020	4695	257	54.74 (48.25-61.85)
Overall	28870	1335	46.24 (43.79-48.79)

U5MR – under-five mortality rate, LB – live births, CI – confidence interval

### Cause of under-five mortality

**Figure 3:**
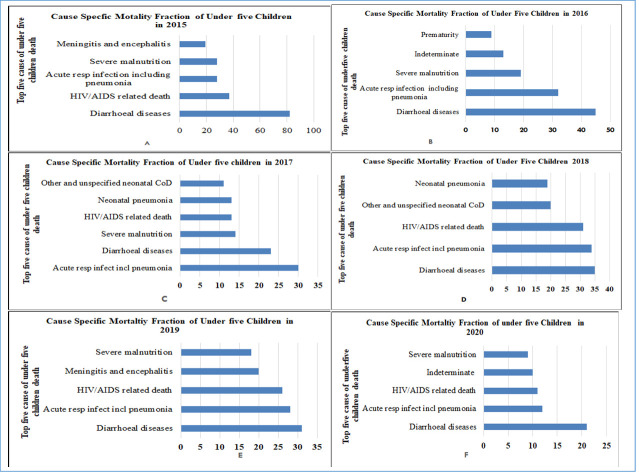
Cause-specific mortality fraction by year among under five children mortality in Kersa HDSS, eastern Ethiopia. COD – cause of death.

**Figure 4:**
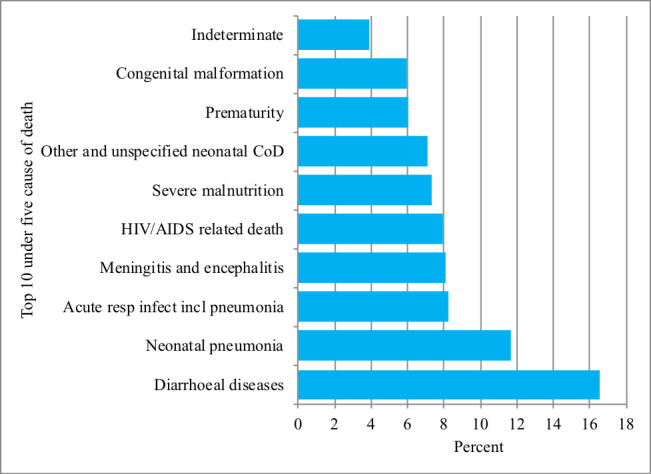
Cause-specific mortality fraction among under five mortality in Kersa HDSS, eastern Ethiopia, 2021. COD – cause of death.

Acute respiratory tract infection including pneumonia, diarrheal diseases and severe malnutrition were among the leading causes of death each year (Figure 3). Overall, diarrheal diseases, acute respiratory tract infection including pneumonia, meningitis and encephalitis, HIV-related deaths and severe malnutrition were the leading causes of death and attributed to more than for 50% of deaths (**Figure 4**).

### Determinants of under-five mortality

Mortality was higher among children born to poor wealth index household (AHR = 1.52; 95% CI = 1.27-1.81), rural residents (AHR = 6.0; 95% CI = 3.65-9.91), born to adolescent mothers (AHR = 1.41, 95% CI = 1.02-1.95), and whose mother never attended ANC (AHR = 1.43, 95% CI = 1.21-1.69). In addition, mortality was higher among both preterm (AHR = 14.1; 95% CI = 9.96-19.89) and post term children (AHR = 1.40; 95% CI = 1.03-1.90) compared to term children. Low birthweight (AHR = 1.74, 95% CI = 1.39-2.18) and big birthweight (AHR = 1.37, 95% CI = 1.05-1.79) were also associated with higher likelihood of early death compared normal birthweight (**Table 3**).

**Table 3:** Determinants of under-five mortality in Kersa DHSS eastern Ethiopia

Variable	CHR (95% CI)	AHR (95% CI)
Residence:		
Harar Town	1	1
Kersa District	6.82 (5.03-9.24)*	6.01 (3.65-9.91)*
Educational status:		
Literate	1	1
Read and/or write	2.01 (1.36-2.96)	1.48 (0.93-2.35)
Neither read nor write	2.10 (1.85-2.38)	1.17 (0.98-1.39)
Occupation:		
House wife	1	1
Daily laborer	0.66 (0.43-1.01)	1.04 (0.62-1.75)
Merchant	0.36 (0.23-0.58)	0.74 (0.32-1.70)
Unemployed	0.86 (0.73-1.02)	0.83 (0.65-1.06)
Paid employee	0.078 (0.02-0.21)	0.48 (0.15-1.57)
Wealth index:		
Poor	1.55 (1.35-1.79)*	1.52 (1.27-1.81)*
Middle	1.41 (1.23-1.63)*	1.27 (1.06-1.53)*
Reach	1	1
Age at first child birth:		
Less than 20 years	1.40 (1.25-1.57)*	1.05 (0.90-1.23)
20 years and above	1	1
Mother’s age (years):		
12-19	1.33 (1.09-1.62)*	1.41 (1.02-1.95)*
20-24	0.89 (0.77-1.03)	0.87 (0.69-1.08)
25-35	1	1
35-49	1.28 (1.12-1.47)*	1.03 (0.85-1.26)
Attended ANC:		
Yes	1	1
No	1.29 (1.12-1.47)	1.43 (1.21-1.69)*
Gravidity:		
1	1	1
2-4	0.93 (0.80-1.09)	0.99 (0.77-1.28)
≥5	1.41 (1.21-1.41)*	0.78 (0.54-1.14)
Term of pregnancy:		
Term	1	1
Preterm	7.15 (5.71-8.95)*	14.08 (9.96-19.89)*
Post term	1.46 (1.19-1.79)*	1.40 (1.03-1.90)*
Birth weight:		
Low	1.93 (1.65-2.25)*	1.74 (1.39-2.18)*
Normal	1	1
Big	0.73 (0.59-0.89)*	1.37 (1.05-1.79)*
Sex of the child:		
Female	1	1
Male	1.07 (0.96-1.19)	1.09 (0.95-1.25)

CHR – crude hazard ratio, AHR – adjusted hazard ratio, ANC – antenatal care, CI – confidence interval, HDSS – Health and Demographic Surveillance System

*P < 0.05.

## DISCUSSION

This study assessed the post-MDG trends, determinants and causes of U5M in a population-based study using verbal autopsy data in eastern Ethiopia. We found that, from 2015 to 2020, U5MR significantly increased while, NMR significantly decreased during the same period. Diarrheal diseases, acute respiratory tract infection including pneumonia, meningitis and encephalitis, HIV-related deaths and severe malnutrition were the leading causes of U5M. U5M was more likely among children born in low economic status households, whose mother never attended ANC, rural residents, born to adolescent mother, preterm and had low birthweight.

Overall, Ethiopia is still among the country with the highest U5MR in Africa [17-19]. Despite the slight reduction in neonatal mortality, which would give hope to the likelihood to achieve the SDG targets, failure to reduce or the increasing trend in overall U5MR requires strong multifaceted and accelerated interventions. One unanticipated finding was the highest U5MR observed in 2019. This could be attributed to the instability in the study area which caused repeated closure of roads to health facilities throughout the year. Generally, this study based on the more reliable and repeated data source clearly pointed out the challenges ahead in Ethiopia to achieve the SDG targets.

Our findings is consistent with the 2019 Ethiopian Mini DHS, which reported U5MR of 55 per 1000 live births [20]. The recent United Nations Inter-agency Group for Child under five mortality estimation also showed high U5M in Ethiopia and highlighted the challenges ahead to meet the SDG goal in this regards [21]. A study which assessed trends of U5M in 31 sub-Saharan African countries estimated the Ethiopian U5MR to be 59 per 1000 live births, classifying Ethiopia among countries with slowest pace of child mortality reduction [17]. Similar finding was also observed in a study conducted in Arbaminch HDSS in Ethiopia which reported 43 death per 1000 live birth [22]. However, the U5MR in this study is slightly higher than the reported 35.6 deaths per 1000 live births in study conducted in Kilite-Awlaelo HDSS site, Tigray Ethiopia [23].

Consistent with other studies, our study showed that many children are dying from communicable diseases: diarrheal diseases and lower respiratory infections [24]. Although the burden of diarrhea among under-five children was reported to decline remarkably in the last two decades [25,26], this does not appear to be the case in this study. One of the issues that emerge from this finding is the major decline in child mortality as well diarrheal disease cause-specific mortality from 2000 to 2016 in Ethiopia is stacked [27]. Reduction of follow-up and support for health extension workers, repeated closure of roads as well as health facilities, and supply chain disruption such as Oral Rehydration Solution (ORS), vaccine, and other lifesaving drugs due to the continued instability in the country in the last few years might explain this observation. Therefore, strengthening and scaling up of behavioral change about importance of personal hygiene and environmental sanitation, child immunization, and strengthening health care seeking is required to reduce the burden of morbidity and mortality attributed to diarrheal diseases.

Children born to adolescent mothers had higher hazard of mortality compared to those 25-34 years old. This is in accord with recent studies indicating up to 4 times increased of risk of death of children born to adolescent mothers compared to those born to young adult mothers [28,29]. Given that more than six in ten women included in this study gave their first birth before 20 years of age, still an unexpectedly high proportion of rural Ethiopian girls are suffering from teenage pregnancy. The observed dramatically worsening survival of children born to adolescent mothers is likely attributed to combination of biological and social factors: poor reproductive maturity and low decision making power of the teens to access sexual and reproductive health services for themselves or their children [29]. There is, therefore, a definite need for reducing adolescent births and its complications as a strategy for addressing the problem of neonatal and child mortality through prevention of early marriage, and ensuring access to quality maternal health services, including use of contraception for delaying teen age pregnancy.

In this study, preterm and low birthweight children were found to be more at risk of dying before celebrating their fifth birthday compared to term birth and normal birth weight, respectively. Increasing risk of mortality with decrease in gestational age at delivery and low birthweight was previously reported [30-32]. Increased risk of death among preterm and low birthweight children born at home was reported due to lack of quality care and treatment for short and long-term health consequences such as hypothermia immediately after birth and in the first days of life [33]. Effect of increased mortality among low-birth-weight due to severe short- and long-term health consequences were also reported elsewhere [19].

The strength of this study was the fact that we followed large cohort of pregnant women from conception to five years after birth. The longitudinal nature of the study enabled us to explore the trends and changes overtime in a stable cohort. However, a number of important limitations should be considered. First, misclassification of cause of deaths using verbal autopsy could over or under-estimate some causes of deaths. In addition, data on cause of deaths was obtained from the closest family member compared to use of physician diagnosis in facility-based studies. This could be prone to bias related with recalling some of the circumstances surrounding the child death.

## CONCLUSION

Against the odds, this study has identified high level of U5MR with noticeable increase in the aftermath of the celebrated MDG success. Despite the early praises for achieving the U5MR reduction as part of the millennium development goals project, this study highlights that Ethiopia is not on track to achieving the 2030 SDG target of U5MR of 25 per 1000 live births. Several factors, ranging from complacence to wide scale insecurity or the persistence of diarrheal diseases, could explain the reasons behind the increasing trends in the U5MR in this predominantly rural setting. As such, commitments to the 2030 targets should consider the multifaceted politico-economic challenges driving the increase in the trends.
